# Supplementary material for: Presence or absence of a prefrontal sulcus is linked to reasoning performance during child development
Source: Brain Struct Funct. 2022 Aug 6;227(7):2543–51. doi: 10.1007/s00429-022-02539-1 (PMC9418286; doi:10.1007/s00429-022-02539-1)
Supplement: Supplementary file 1 — Supplementary file1 (PDF 13389 kb) [file 429_2022_2539_MOESM1_ESM.pdf]

Supplementary Materials for

Presence or absence of a prefrontal sulcus is linked to reasoning performance during child development

Ethan H. Willbrand, Willa I. Voorhies, Jewelia K. Yao,
Kevin S. Weiner*, Silvia A. Bunge
*Corresponding author. Email: kweiner@berkeley.edu

This PDF file includes:

Supplementary Results
Supplementary Figures 1-5
Supplementary Table 1

Supplementary Results

Pimfs surface area explains marginally more variance than age alone

Pimfs morphology was also behaviorally relevant when examined with a continuous metric (total surface area of the sulcus), albeit to a lesser degree than the discrete metric of presence/absence of a pimfs component. Specifically, a linear regression (with age included as a covariate), revealed that the total surface area of the left pimfs (ß = 0.01, t = 2.35, p = .022) was positively associated with reasoning (Supplementary Fig. 4); this relationship was marginal (ß = 0.01, t = 1.85, p = .068) in the right hemisphere. Whereas the discrete models examining sulcal components explained *significantly* more variance in reasoning than age alone, this model only explained *marginally* more variance than age alone (pimfs: $R^2_{adj} = 0.49$, $p < .001$; age: $R^2_{adj} = 0.46$, $p < .001$; model comparison: $p = .071$). A repeated K-fold (5-fold, 10 repeats) and leave-one-out cross-validation (looCV) confirmed that the predictiveness of the total pimfs surface area model (*5-fold*: $R^2 = 0.51$, RMSE = 4.33; *looCV*: $R^2 = 0.45$, RMSE = 4.45) was only slightly better than age alone (*5-fold*: $R^2 = 0.49$, RMSE = 4.40; *looCV*: $R^2 = 0.43$, RMSE = 4.50). Further, when normalizing left pimfs surface area by the total surface area of the PFC, the relationship was marginal (p = .075). Thus, from our analyses, the presence or absence of pimfs-v was more directly linked to reasoning than total pimfs surface area, which was related to PFC surface area more broadly.

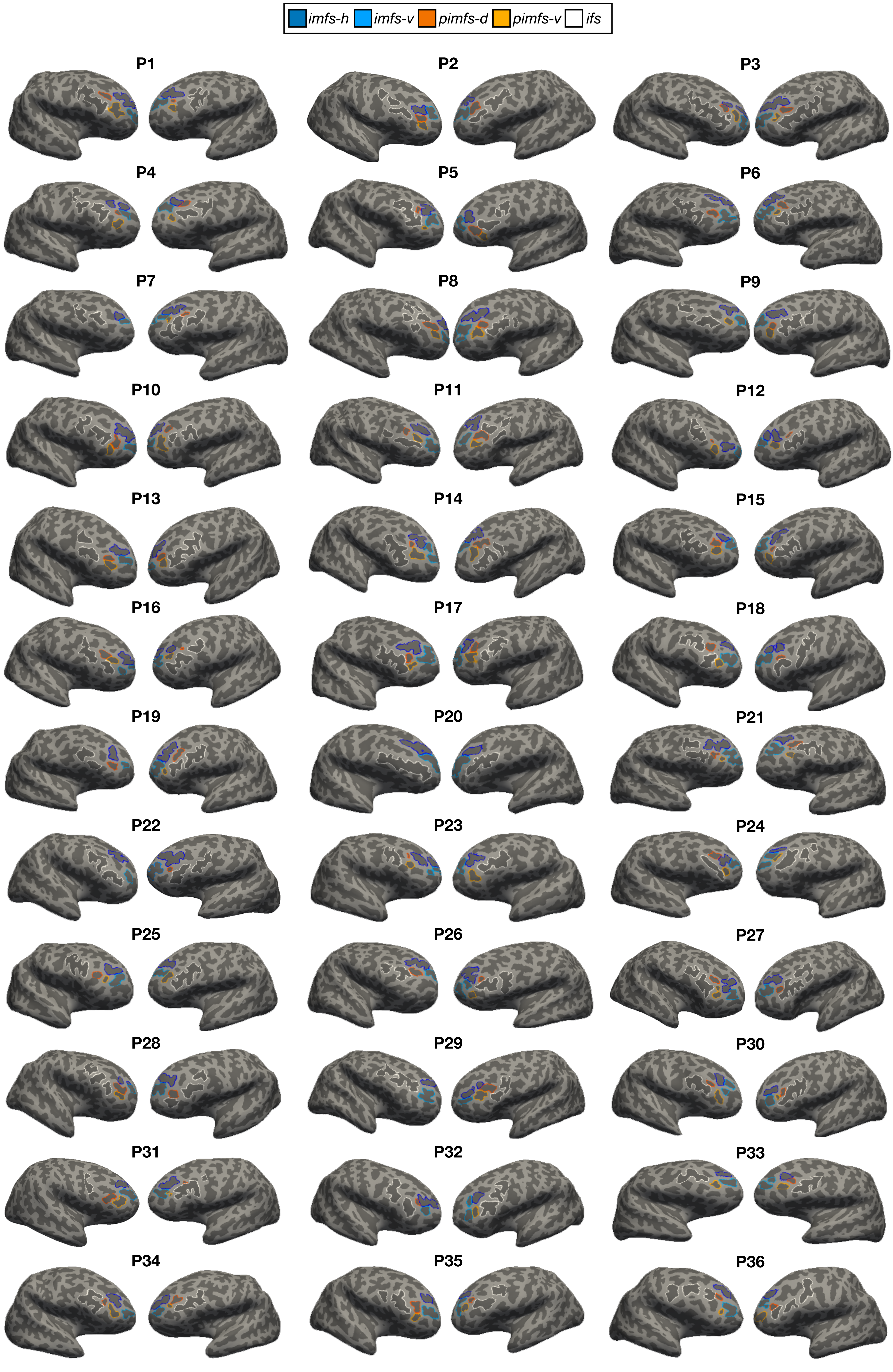
imfs-h
imfs-v
pimfs-d
pimfs-v
ifs
P1
P2
P3
P4
P5
P6
P7
P8
P9
P10
P11
P12
P13
P14
P15
P16
P17
P18
P19
P20
P21
P22
P23
P24
P25
P26
P27
P28
P29
P30
P31
P32
P33
P34
P35
P36

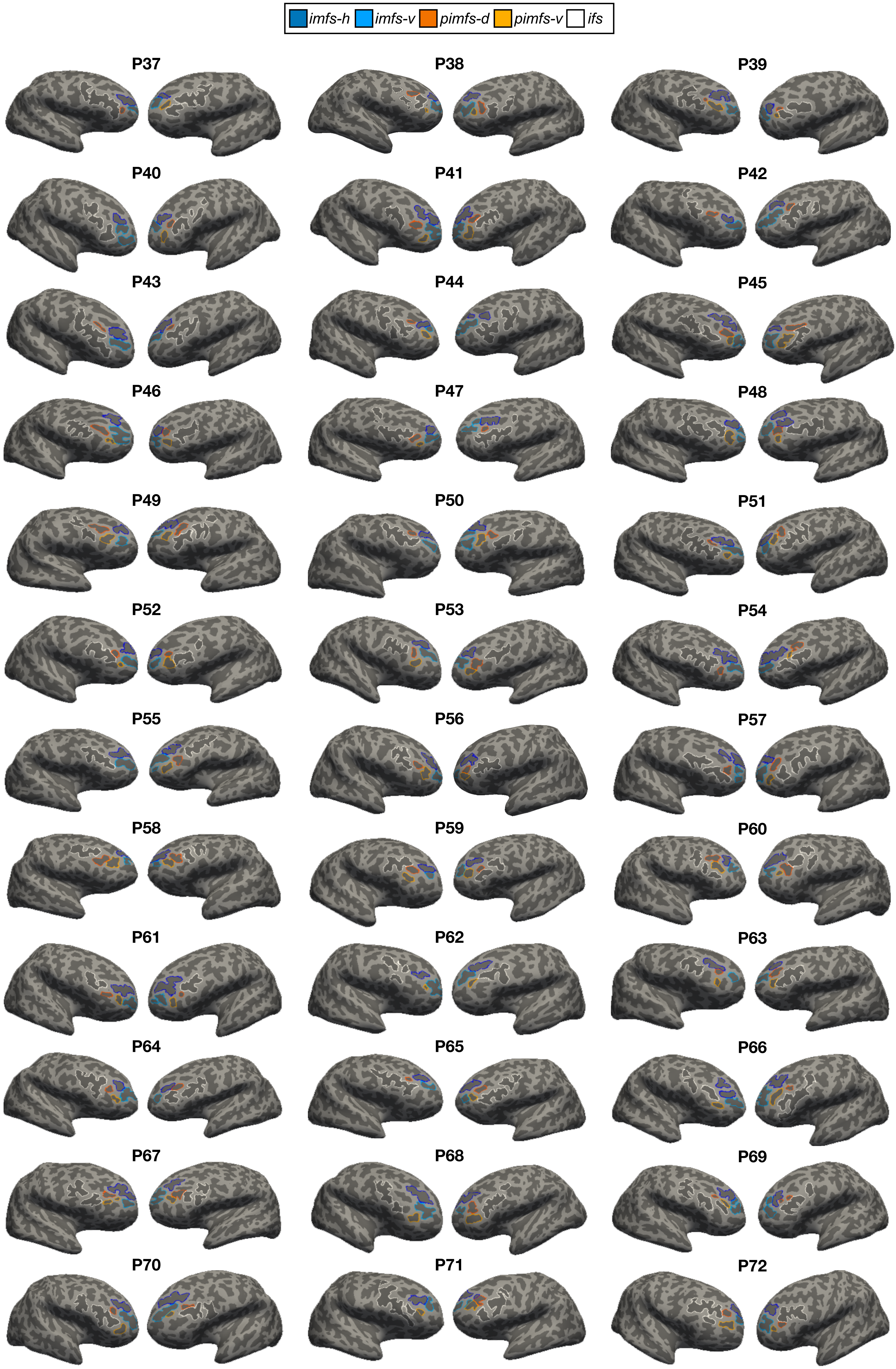
imfs-h
imfs-v
pimfs-d
pimfs-v
ifs
P37
P38
P39
P40
P41
P42
P43
P44
P45
P46
P47
P48
P49
P50
P51
P52
P53
P54
P55
P56
P57
P58
P59
P60
P61
P62
P63
P64
P65
P66
P67
P68
P69
P70
P71
P72

Supplementary Figure 1. Manual sulcal labels in the left and right hemispheres of each participant (N=72). As in Figure 1A, each sulcus is displayed on the inflated cortical surface (surfaces are not to scale) in FreeSurfer 6.0.0 and is colored according to the key at the top. Sulci were defined according to the most recent atlas and criteria by Petrides (2013, 2019; Materials and Methods). All hemispheres have the horizontal (imfs-h; dark blue) and ventral (imfs-v; light blue) intermediate frontal sulci and inferior frontal sulcus (ifs; white). The para-intermediate frontal sulcus (pimfs; orange) is more variable: participants can have zero, one (dorsal (dark) or ventral (light)), or two components (dorsal and ventral).

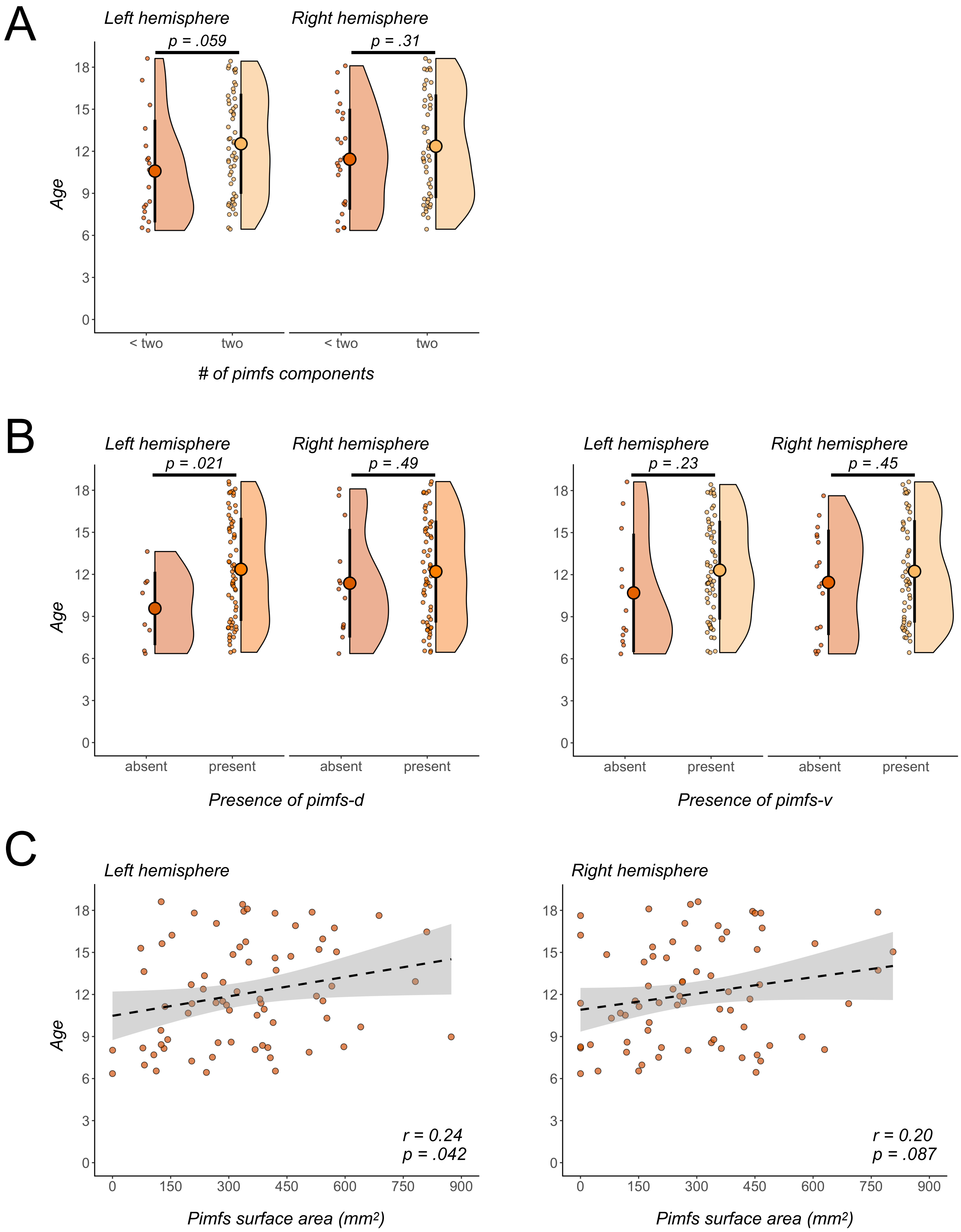

Supplementary Figure 2. Relationship between age and para-intermediate frontal sulcus morphological metrics. (**A**). Raincloud plot (Allen et al. 2021) depicting age as a function of the number of para-intermediate frontal sulcus (pimfs) components in the left and right hemispheres using the whole sample (N = 72). The large dots and error bars represent the mean±std reasoning score and the violin shows the kernel density estimate. The smaller dots indicate individual participants. These features are colored (dark and light orange) to distinguish between the two groups. (**B**). Same format as (A), except for the presence/absence of the pimfs-d (*Left*) and pimfs-v (*Right*). (**C**). Scatterplot visualizing age as a function of left and right pimfs surface area (mm^2). The best fit line, ±95% confidence interval, and correlation coefficient (r) are included. The smaller dots represent individual participants (N = 72). Despite there being correlations between age and some pimfs features, these collinearities did not affect the model results (see Materials and Methods).

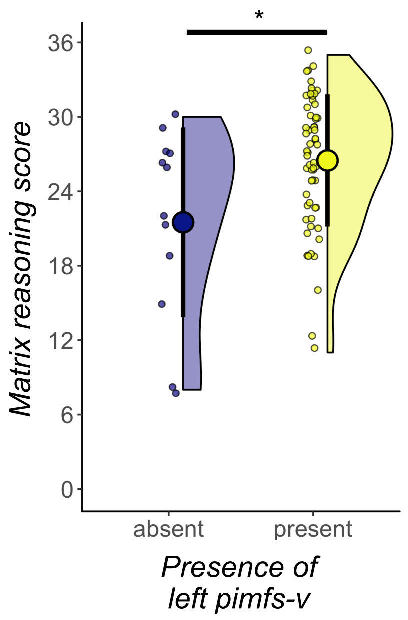

Supplementary Figure 3. The presence of the ventral para-intermediate frontal sulcus is related to reasoning (whole sample). Raincloud plot (Allen et al. 2021) depicting reasoning score as a function of the presence of the ventral para-intermediate frontal sulcus (pimfs-v) component in the left hemisphere using the whole sample (N = 72). The large dots and error bars represent the mean±std reasoning score and the violin shows the kernel density estimate. The smaller dots indicate individual participants. These features are colored (blue and yellow) to distinguish between the two groups. After controlling for age, those with the pimfs-v in the left hemisphere (*p = .027) had better reasoning scores than those without.

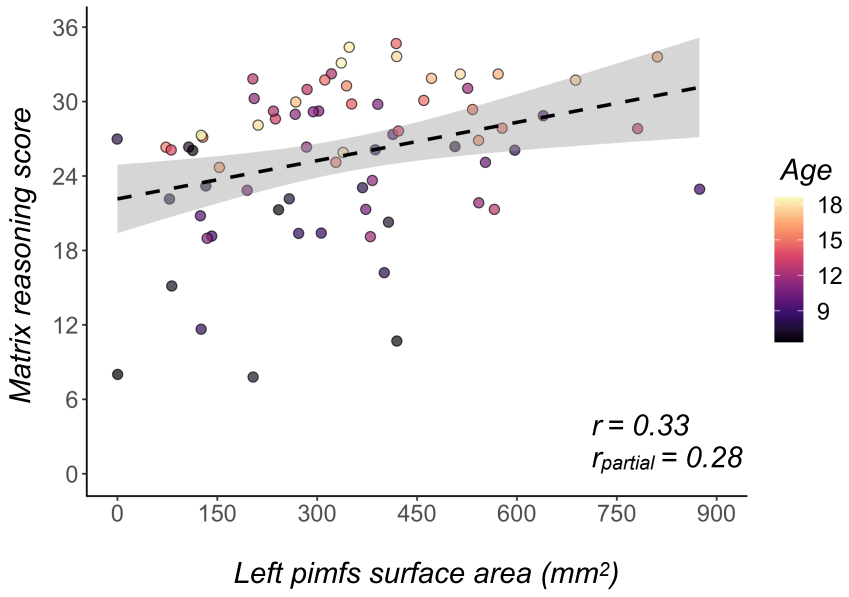

Supplementary Figure 4. Pimfs surface area explains marginally more variance than age alone. Scatterplot visualizing reasoning scores as a function of left para-intermediate frontal sulcus (pimfs) surface area (raw, in mm^2), controlling for age. The best fit line, ±95% confidence interval, correlation coefficient (r), and $r_{partial}$ from the regression are included. The smaller dots represent individual participants, colored by age (darker = younger; lighter = older). In the left hemisphere, the surface area of the pimfs is positively associated with reasoning (p = .022), even after controlling for age. Nevertheless, whereas the discrete models examining sulcal components explained significantly more variance in reasoning than age alone, a linear model explained marginally more variance than age alone (pimfs: R^2_{adj} = 0.49, p < .001; age: R^2_{adj} = 0.46, p < .001; model comparison: p = .071; Supplementary Results).

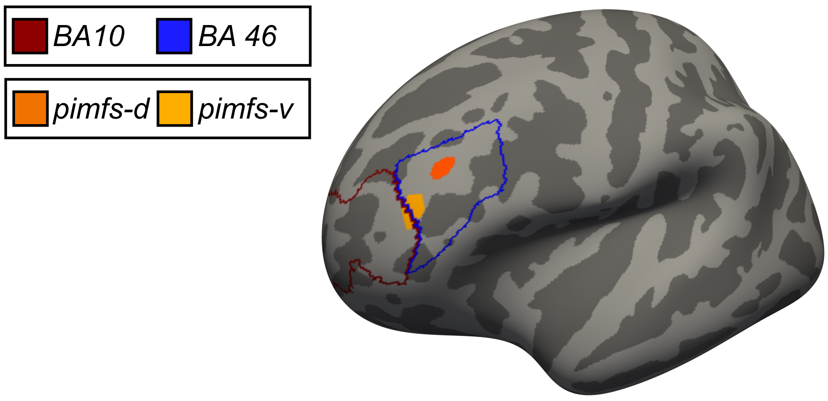

Supplementary Figure 5. The para-intermediate frontal sulcus relative to Brodmann Areas in LPFC associated with reasoning. Left hemisphere inflated fsaverage surface with putative para-intermediate frontal sulcus (pimfs) components and Brodmann Areas (BA) overlayed onto the surface. BAs and pimfs components are color-coded according to the key. Here, the pimfs-v (light orange) likely serves as a transition zone (border) between BA 46 (blue) and BA 10 (maroon). Notably, BA 10/46 overlaps with functionally-defined rostrolateral prefrontal cortex, a sub-region of LPFC implicated in reasoning abilities (for review see Vendetti and Bunge 2014).

	Count
Racial categories	
American Indian/Alaskan Native	0
Asian/Native Hawaiian/Other Pacific Islander	5
Black or African American	4
White	47
More Than One Race	14
Unknown or Not Reported	2
Ethnic categories	
Hispanic or Latino	11
Not Hispanic or Latino	59
Unknown or Not Reported	2
Highest degree earned by parent/guardian	
High School/GED	8
Vocational	1
Associate degree	6
Bachelor's degree	18
Master's degree	15
Doctorate	4
Professional	3
Other	3
None of the above (less than high school)	1
Unknown or Not Reported	13
Total household income	
$16,000-$24,999	2
$25,000-$34,999	3
$50,000-$74,999	6
$75,000-$99,999	8
$100,000-$199,999	27
Over $200,000	5
Unknown or Not Reported	21

Supplementary Table. 1. Demographic and socioeconomic information of the child/adolescent sample (N = 72). All information is parent/guardian reported.
